# Health and social care professionals’ awareness and implementation of NICE guidelines on self-harm: a rapid review of the literature

**DOI:** 10.1136/bmjopen-2024-093883

**Published:** 2025-08-19

**Authors:** Gill Lever, Dawn Dowding, Dharman Jeyasingham, Christopher J Armitage

**Affiliations:** 1 NIHR Greater Manchester Patient Safety Research Collaboration, University of Manchester, London, England, UK; 2 University of Manchester, Manchester, England, UK; 3 Manchester Centre for Health Psychology, Coupland 1 Building, University of Manchester and NIHR Greater Manchester Patient Safety Research Collaboration, Manchester, England, UK

**Keywords:** Suicide & self-harm, PUBLIC HEALTH, Implementation Science

## Abstract

**Abstract:**

**Objectives:**

To identify the factors influencing professionals’ implementation of the National Institute for Health and Care Excellence (NICE) guidelines on self-harm.

**Design:**

A rapid review evidence synthesis

**Data sources:**

Five electronic databases (ASSIA, CINAHL, EMBASE, MEDLINE, PsycINFO) and five indexing databases (Science Citation Index Expanded (SCIE), Social Sciences Citation Index (SSCI), Arts and Humanities Citation Index (AHCI), Emerging Sources Citation Index (ESCI) and Conference Proceedings Citation Index (CPCI)), using the Web of Science platform, were searched in December 2023 and repeated in July 2024.

**Eligibility criteria:**

We included quantitative and qualitative studies that investigated professionals’ knowledge and implementation of NICE guidelines on self-harm, that were in English language and published between 2004 and July 2024.

**Data extraction and synthesis:**

One reviewer used standardised methods to search, screen, select, quality assess and synthesise the included studies, to accelerate the review. Quality assessment was conducted using the Mixed Methods Appraisal Tool. Data were extracted and synthesised thematically using NICE guidance implementation priorities.

**Results:**

The review included 10 studies. Six were conducted in accident and emergency (A&E) settings, two in general practice, one in a burns and plastic surgery hospital department and one involved cross-sectoral health professionals. Key findings indicate that awareness and implementation of self-harm guidelines is low among health professionals. Systemic barriers include lack of staff training, negative staff attitudes towards people who self-harm and lack of resources.

**Conclusions:**

There is a need to develop and implement regular training on self-harm, incorporating NICE guidance and measures, to integrate knowledge and mobilise practice changes. Further research into the implementation of NICE guidelines in children who self-harm is needed, and in a wider variety of health and social care settings. The absence of studies from the social care sector into professionals’ awareness and implementation of NICE guidelines on self-harm is a key limitation.

**STRENGTHS AND LIMITATIONS OF THIS STUDY**

- This rapid review synthesised the results of 10 studies, identified from searches of five electronic databases and five indexing databases (using the Web of Science platform), assessed their quality and methodological rigour, using a standardised appraisal tool and adheres to the PRISMA (Preferred Reporting Items for Systematic Reviews and Meta-Analyses) recommendations for reporting this rapid review.
- Barriers and facilitators to implementation across sectors and settings were identified, reviewed and mapped against the eight key priorities for implementation of the NICE (National Institute for Health and Care Excellence) 2004 guidelines for self-harm.
- A major limitation of this rapid review is that a single reviewer searched, screened, selected, quality assessed and analysed the studies.
- The structured approach to data synthesis limited the depth and richness of the analysis and may have excluded nuanced barriers and facilitators to guideline implementation.
- Omission of non-English literature and narrow search strategy to identify implementation may not have captured all relevant articles.

## Introduction

Self-harm, characterised by self-poisoning or injury, regardless of intent or motivation,^1^ is a significant health and social care problem in the UK. The prevalence of self-harm has increased substantially over the last 20 years.^2^ The onset of self-harm is typically between 12 and 14 years^3^ although evidence of younger children increasingly engaging in self-harming behaviours is emerging.^4^ A recent cohort study reported that one in five adolescents, in the UK, has self-harmed.^5^

The National Institute for Health and Care Excellence (NICE) produces guidance to promote and protect good health, reduce health inequalities and improve the quality of care and services.^1^ NICE first published guidelines on the management and prevention of self-harm in 2004. Emphasising that the experience of care for people who self-harm is ‘often unacceptable’,^6^ the guideline set out key priorities for implementation and recommendations for practice and research, on the short-term physical and psychological management and secondary prevention of self-harm in primary and secondary care. NICE guidelines on the longer-term psychological treatment and management of both single and recurrent episodes of self-harm management followed in 2011, addressing all health and social care professionals who encounter people who self-harm.^7^ Both sets of guidance highlighted respectful and non-judgemental ways of working with people who self-harm, assessment of risks and needs/psychosocial assessment and interventions for self-harm, as key priorities for implementation.^6 7^ Despite the dissemination of the NICE guidelines across primary and secondary care health services, professional organisations and statutory bodies and the Royal Colleges, poor practice in the care and treatment of self-harm has persisted.^8^,^13^ Demographic inequalities have also endured with regional variations in hospitalisation, resulting from self-harm, correlating with socioeconomic deprivation.^14 15^ Subsequent systematic reviews of the experiences of people who self-harm have found that they report largely negative experiences of care from clinical health services, in the priority implementation areas.^2^ Much has been written about stigmatising attitudes and microaggressions from staff,^10 12 16 17^ and rushed, generic tick-box questions being used, instead of comprehensive risk and psychosocial assessments.^17^,^19^ There is some evidence of psychopharmacology being routinely endorsed as a first-line treatment option, without consideration of psychological intervention and the needs and preferences of the patient.^16 19^ These findings indicate that the publication of NICE guidelines has not had the impact of improvements in care for self-harm patients.^2^

Following a Healthcare Safety Investigation Branch report emphasising the confusion inherent in the presence of separate guidelines for short-term and long-term management of self-harm,^20^ NICE guidelines on self-harm were updated in 2022, into a single guideline: Self-harm: assessment, management and preventing recurrence.^1^ The updated guidance acknowledges the increasing prevalence of self-harm across ages and the heterogeneity of services and settings that are managing self-harm episodes, extending their recommendations for practice to all sectors that work with people who have self-harmed.

Although the guidelines offer renewed impetus to improve the standard and quality of care provided to people who self-harm, previous research has demonstrated that professional awareness of government policies and guidelines can be low.^21^,^23^ Therefore, implementation across sectors is likely to be dependent on the dissemination of interdisciplinary staff training, regular protected and reflexive supervision and potentially new models of care and updated, professional competency frameworks.^24^ Given these concerns, a rapid review was conducted to identify factors influencing the implementation of NICE guidelines on self-harm, across services and settings.

### Aims and objectives

- To estimate the prevalence of health and social care professionals’ awareness and implementation of NICE guidelines on self-harm.
- To identify the facilitators and barriers to the implementation of NICE guidelines on self-harm.
- To provide insights and recommendations on the most effective methods for increasing professionals’ compliance with NICE guidelines on self-harm.

## Methods

A rapid evidence synthesis is a time-sensitive, pragmatic and efficient means of summarising and contextualising the best available research evidence. It has increased relevance to decision-making, being time-sensitive, while retaining rigour and quality assessment. Rapid evidence syntheses are well-placed to facilitate imminent and innovative further research and intervention, through timely identification of gaps in the evidence base.^25 26^ In this research, it is instrumental in ensuring prompt decision-making concerning salient research directions to provide potential innovations that could improve outcomes for self-harm practice. While adhering to the core principles of the traditional systematic review, literature searches were restricted to English language only. Single coding of the search and selection strategies, quality assessment and thematic analysis was used to accelerate the literature synthesis.

### Search strategy

A search strategy was devised to identify studies investigating professionals’ knowledge and implementation of NICE guidelines on self-harm across services and sectors. An iterative process generated search terms for each component of the model, which were combined in the final search.

The NICE (2022) definition of self-harm, which emphasises the intentionality of self-poisoning or injury, irrespective of motivation, incorporates attempted suicide.^1^ This guided keywords for the search. Search terms included ‘NICE guidelines’ and ‘guidance’, ‘self-harm’, ‘DSH’, ‘self-injury’, ‘non-suicidal self-injury’, ‘self-mutilation’, ‘self-poison’, ‘parasuicide’ and ‘suicide’. Search terms for implementation included ‘implement’, ‘compliance’, ‘adherence’, ‘knowledge’, ‘barrier’, ‘facilitation’, ‘enable’ and ‘challenge’. Wildcards and Boolean operators (AND, OR) were used to confine or expand the search.

Searches were conducted by a single reviewer (GL). Five electronic databases (ASSIA, CINAHL, EMBASE, MEDLINE, PsycINFO) and the platform, Web of Science, using five indexing databases (Science Citation Index Expanded (SCIE), Social Sciences Citation Index (SSCI), Arts and Humanities Citation Index (AHCI), Emerging Sources Citation Index (ESCI) and Conference Proceedings Citation Index (CPCI)) were searched for relevant published literature in December 2023. Additionally, Google Scholar was also used to identify relevant studies, searching the first 10 pages of results, generated from keywords.

Handsearching of references of full-text articles retrieved was also performed. Searches were repeated in July 2024, where 13 extra studies were identified but not included in the review. The updated search generated 298 studies. The full search strategy for each database is provided in online supplemental appendix 1.

### Inclusion criteria

Search parameters for inclusion were any original quantitative, qualitative or mixed-methods study design. There were no restrictions on sample size or peer review status. Searches were limited to English language publication, between 2004 and July 2024. The cut-off at 2004 ensured that only studies conducted following publication of NICE guidelines on self-harm were incorporated into the review.

### Exclusion criteria

Studies not providing original results: systematic reviews, meta-analyses or general literature reviews. Editorials and opinion articles and research protocols were excluded. Studies conducted before 2004, and studies of non-English language publication were also ineligible.

### Study selection

The selection process was conducted stepwise, according to Polit and Beck’s^27^ selection strategies.^27^ Studies included in the final analysis that met the inclusion criteria were studies that had focused on an assessment of NICE guidelines usage and conformed to NICE guidelines’ definition of self-harm. This involved initially selecting articles with relevant titles and reviewing their abstracts.

### Data extraction, transformation and analysis

A single reviewer, GL, performed data extraction from the studies, using an Excel spreadsheet. A framework matrix of the eight key priorities for implementation of the NICE 2004 guidelines for self-harm was constructed to categorise barriers and facilitators. Using the convergent integrated approach recommended by the Joanna Briggs Institute Mixed Methods Review Methodology Group,^28^ for integrating qualitative and quantitative methodology, a single reviewer, GL, converted the quantitative data into textual descriptions (qualitative data). Although most studies^8^ were quantitative, findings were codified into qualitative data, as this method is less prone to error than quantifying qualitative data.^28^ The converted textual data was combined with the qualitative data extracted from the two qualitative studies and concurrently analysed. Coding was performed by a single reviewer (GL) and grouped into generated themes that were revised and refined through the process of reflection and referring back to the original articles. GL tabulated data on the included articles’ study characteristics. Extracted data included: authors, year of publication, data collection period, research design and methodology, setting, population and their characteristics, sample size, inclusion/exclusion criteria and key findings related to awareness and implementation rates of the NICE guidelines on self-harm and the barriers and/or facilitators to implementation.

### Quality assessment

The 10 included articles were quality assessed by a single reviewer (GL) according to the Mixed Methods Approval Tool (MMAT) V.2018 criteria.^29^ As the review questions are complex, comprising qualitative and quantitative elements, this appraisal tool was selected to enable concomitant quality appraisal of mixed methods studies and qualitative and quantitative studies.^30^ The MMAT comprehensively covers study design, sampling, data collection, data analysis and replicability.^29^

### Patient and public involvement

Patients and/or the public were not involved in the design, or conduct, or reporting, or dissemination plans of this research.

## Results

**Figure 1:**
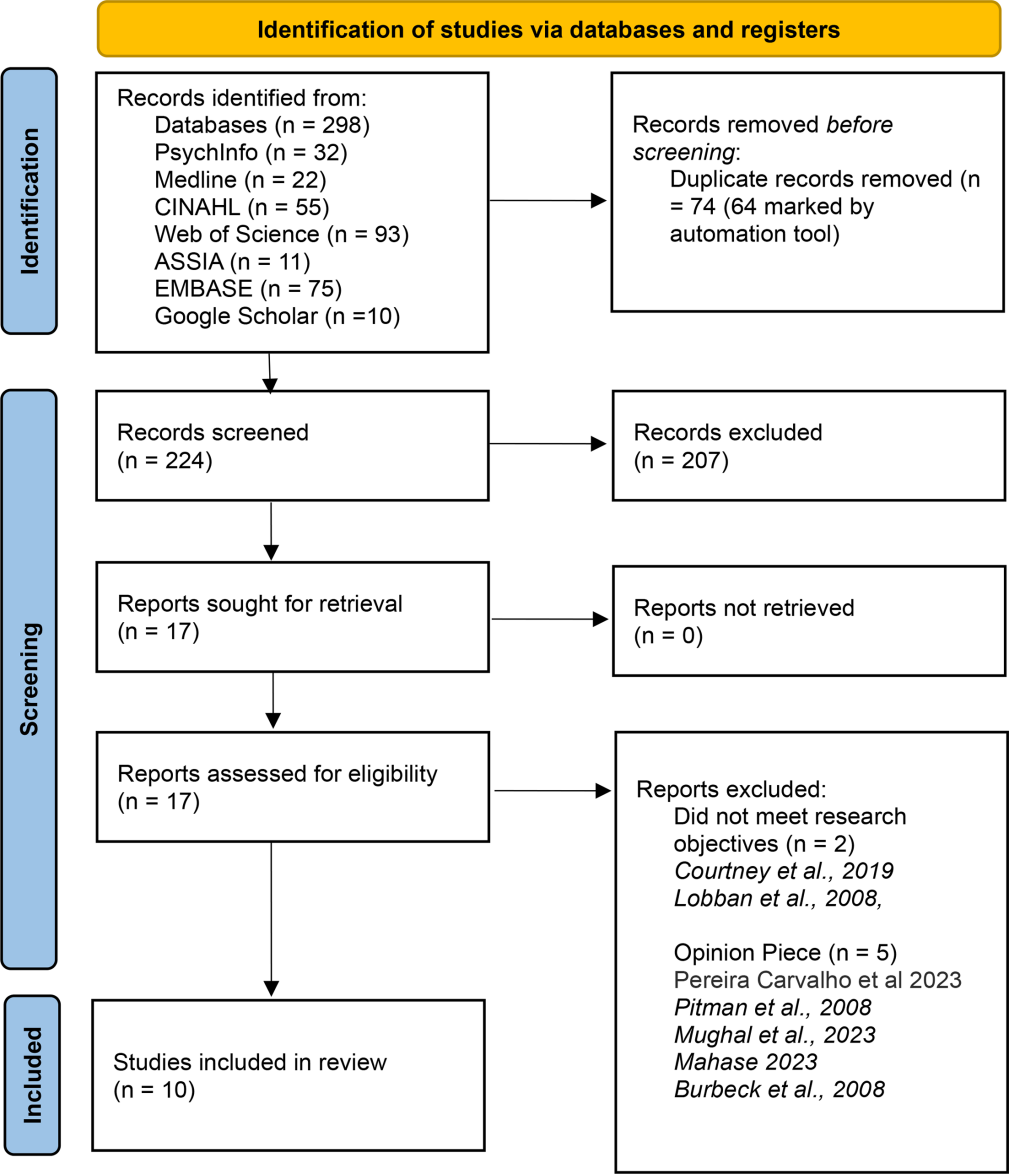
Preferred Reporting Items for Systematic Reviews and Meta-Analyses flow chart of the search and screening process.

The search strategy generated a total of 298 publications. 74 of these were duplicates, which were then excluded. 224 titles and abstracts were screened by a single reviewer (GL), based on the inclusion criteria and 17 articles were retrieved for full text review. Seven articles were subsequently excluded; two due to not meeting the research objectives and five being opinion pieces. Citations for the articles excluded, due to ineligibility, at the stage of full article review, are listed in online supplemental appendix 2. This resulted in 10 articles that met the inclusion criteria. A summary of the selection process is presented in figure 1.

### Mixed Methods Approval Tool V.2018

The MMAT quality assessment (refer to online supplemental appendix 3) resulted in six studies being graded as high quality 31,36 and four graded as moderate quality,^37^,^40^ due to high or unclear risk of non-response bias, lack of reliability and validity testing of measures and presence of confounding bias. No studies were excluded based on quality. The results of the MMAT are considered when interpreting the results and to inform the discussion.

### Methodological quality

Several of the studies have representation and generalisability of results impacted by non-response bias. The study of major trauma centres and networks (40) yielded a non-response rate of 52%, (n=16/31) despite the efforts of the authors, who followed up correspondence to engage in the study three times with centres that did not reply. In the cross-sectoral study of health professionals,^39^ routing errors resulted in around 200 eligible participants not responding to a core question concerning the implementation of NICE guidelines. The only study within the review that captured service user experiences showed mainly positive experiences from patients. However, it was hampered by a low-response rate (14/43).^35^ The paper does not describe how the survey was carried out or potential factors affecting low response rates. The questionnaire does not capture the method of self-harm of patients, responding to their treatment in an accident and emergency department. This is an important omission, as staff attitudes towards self-harm patients have been shown to differ between self-laceration and self-poisoning, with the former garnering less compassion.^41^ The quality-of-service patients receive may therefore be affected by self-harm method.

None of the 10 studies reviewed assessed professionals’ use of the NICE (2022) guidelines: Self-harm: assessment, management and preventing recurrence. (1) All studies used the NICE (2004) guidelines: Self-harm: the short-term physical and psychological management and secondary prevention of self-harm in primary and secondary care.^6^ (Refer to box 1 for descriptions of the priority areas of NICE guidance that are assessed in this review).

Box 1 **Priority Areas Recommended in NICE (National Institute for Health and Care Excellence, 2004) Self-Harm: The Short-Term Physical and Psychological Management and Secondary Prevention of Self-Harm in Primary and Secondary Care**

**Priority 1: Respect, understanding and choice**

People who have self-harmed should be treated with the same care, respect and privacy as any patient. In addition, healthcare professionals should take full account of the likely distress associated with self-harm.

**Priority 2: Staff training**

Clinical and non-clinical staff who have contact with people who self-harm in any setting should be provided with appropriate training to equip them to understand and care for people who have self-harmed.

**Priority 3: Activated charcoal**

Ambulance and emergency department services whose staff may be involved in the care of people who have self-harmed by poisoning should ensure that activated charcoal is immediately available to staff at all times.

**Priority 4: Triage**

All people who have self-harmed should be offered a preliminary psychosocial assessment at triage (or at the initial assessment in primary or community settings) following an act of self-harm. Assessment should determine a person’s mental capacity, their willingness to remain for further (psychosocial) assessment, their level of distress and the possible presence of mental illness.

**Priority 5: Treatment**

People who have self-harmed should be offered treatment for the physical consequences of self-harm, regardless of their willingness to accept psychosocial assessment or psychiatric treatment.

**Priority 6: Assessment of needs**

All people who have self-harmed should be offered an assessment of needs, which should be comprehensive and include evaluation of the social, psychological and motivational factors specific to the act of self-harm, current suicidal intent and hopelessness, as well as a full mental health and social needs assessment.

**Priority 7: Assessment of risk**

All people who have self-harmed should be assessed for risk: this assessment should include identification of the main clinical and demographic features known to be associated with risk of further self-harm and/or suicide, and identification of the key psychological characteristics associated with risk, in particular depression, hopelessness and continuing suicidal intent.

**Priority 8: Psychological, psychosocial and pharmacological interventions﻿**

Following psychosocial assessment for people who have self-harmed, the decision about referral for further treatment and help should be based upon a comprehensive psychiatric, psychological and social assessment, including an assessment of risk, and should not be determined solely on the basis of having self-harmed.

### Characteristics of included studies

All studies were based in the UK and Ireland. Of the 10 studies included in the review, 2 of the studies were qualitative^31 32^ and 8 were quantitative.^33^,^40^ The included studies all collected data between May 2004 and August 2019 and were published between 2007 and 2023. Services and settings included in the studies were general practice^31 32^ and hospital accident and emergency,^33^,^3740^ a burns and plastic surgery department^38^ and cross-sectoral health professionals.^39^ Study characteristics and main findings are displayed in table 1.

**Table 1:** Study characteristics and awareness and implementation rates of NICE (2004) guidance on self-harm

First author name	Year	Data collection period	Country	Research design	Setting	Sample size	Awareness rate %	Implementation rate %
Cracknell	2015	2012–2014	England	Quantitative repeat audit	A&E	150 patients	NA	14
Cooper	2008	Jan–Feb 2006	England	Quantitative audit	A&E	93 patients	NA	51
Heyward-Chaplin	2018	April 2019	England	Quantitative online survey	Burns and plastic surgery	59 staff	12	NA
Hughes	2007	May 2004–2005	England	Quantitative repeat audit	A&E	203 patients	NA	5.8
Jones	2007	Spring 2007	Wales	Quantitative audit and survey	A&E	50 patients 124 staff	NA	50
Leather	2020	April 2019	UK	Quantitative online survey	Cross-sectoral	1020 staff	24	44
Leather	2022	April–May 2019	UK	Qualitative semi-structured interview	General practice	21 staff	NA	NA
Leather	2023	April–May 2019	UK	Qualitative semi-structured interview	General practice	12 staff	NA	NA
Mullins	2010	Jan–Dec 2006	Ireland	Quantitative audit	A&E	834 staff	NA	59
Stallard	2022	July–Aug 2019	Scotland England	Quantitative audit survey	A&E	15 staff	NA	53

A&E, accident and emergency; NICE, National Institute for Health and Care Excellence.

Two studies reported awareness rates of NICE guidelines on self-harm among professionals, measured by questionnaire, asking participants of their awareness of the UK NICE guidelines. Both cited low awareness rates of 12% (n=7) (35) and 24% (n=248), respectively.^39^

However, it is notable that the studies differed in their definition and calculation of awareness rates. While both studies asked participants if they were aware of the UK NICE guideline ‘Self-harm in over 8s: short-term management and prevention of recurrence’, one study reported awareness rates based on participants reporting being ‘aware’ rather than ‘not aware’ or ‘unsure’.^38^ In this study, awareness was indicative of knowledge of the guidelines’ existence. A follow-up question asking if participants had read the guidelines revealed that just 5% (n=3) of the sample reported having done so. Whether or not participants knew the content of the guidelines well was not measured. The other study measured awareness rates on a five-point scale (from ‘never heard of’ to ‘know very well’).^39^ Although 86% of the sample (n=873) had heard of the NICE guideline for self-harm, respondents who were categorised in the analysis as being ‘aware of the guidelines’ were those who knew ‘a fair amount’ about the guidelines or knew them ‘very well’.

How the implementation of the guidelines was calculated varied across the studies. None of the studies objectively examined implementation of all the eight priorities in the guidance, and one study focused solely on triage.^33^ Implementation of NICE guideline recommendations ranged between 5.8% and 59% (mean=39.59, SD 19.34),^33^,^3739 40^ as measured by recorded adherence to psychosocial assessment in patient notes for four studies,^3335^,^37^ a survey of estimated compliance of psychosocial assessment,^40^ recorded risk assessment implementation rates in patients notes^34^ and practitioner estimated general adherence to all aspects of NICE guidance, rated on a percentage scale.^39^ Following the development of proformas and prompts designed to raise compliance with NICE guidance recommendations, some elements of documentation of alignment rose as high as 97%, for example, for the recording of capacity to consent in patient notes.^34^ Documentation of full mental health and social needs assessments ranged from 14% to 59% (mean=45.46, SD=16.04)^3335^,^37 40^ across studies reporting quantitative data on this priority. However, rates of compliance with NICE were declared because of tasks being checked and documented in patient notes.^36^ There was no examination of the quality of the assessment, for example, how the risk of suicide or recurrent self-harm had been assessed. None of the studies considered patient involvement in the assessment when calculating rates of implementation. NICE guidance on the psychosocial assessment encourages joint decision-making and recommends that service users and assessors both read and agree on the written assessment of needs. Where there is significant disagreement, the service user should be offered the opportunity to write their disagreement in the notes.^6^ As patient involvement in the assessment was not explored in any of the studies, it is concluded that the higher rates of compliance recorded based on a psychosocial assessment being documented do not represent true adherence to NICE recommendations, and that there is no evidence that patients are routinely involved in decision-making and reviewing their data. Furthermore, NICE (2004) advises that the decision to refer for further assessment and/or treatment or to discharge the service user should be taken jointly by the service user and the healthcare professional, whenever this is possible.^6^ However, by not examining this, the included studies indicate that this was another aspect of the assessment that was not included when calculating implementation rates of the NICE guidelines.

Barriers and facilitators to implementation were identified, reviewed and mapped against the eight key priorities for implementation of the NICE 2004 guidelines for self-harm displayed in table 2. The two qualitative studies^31 32^ included in this review provided data on barriers and facilitators, derived through thematic analysis. The results were analysed again and coded and themed with the cumulative data of all 10 studies.

**Table 2:** Studies reporting on NICE priority areas. Priority Areas Recommended in NICE (2004) Self-Harm: The Short-Term Physical and Psychological Management and Secondary Prevention of Self-Harm in Primary and Secondary Care

	P1	P2	P3	P4	P5	P6	P7	P8
Cracknell^37^		●	●	●	●	●	●	●
Cooper *et al*^33^				●		●		
Heyward-Chaplin *et al*^38^	●	●			●			
Hughes and Kosky^34^		●		●			●	
Jones and Avies-Jones^35^	●	●	●	●		●	●	
Leather *et al*^39^		●					●	
Leather *et al*^31^							●	●
Leather *et al*^32^		●		●		●	●	●
Mullins *et al*^36^		●		●		●	●	
Stallard *et al*^40^	●			●		●		●

Priority 1: Respect, understanding and choice

Priority 2: Staff training

Priority 3: Activated charcoal

Priority 4: Triage

Priority 5: Treatment

Priority 6: Assessment of needs

Priority 7: Assessment of risk

Priority 8: Psychological, psychosocial, and pharmacological interventions

NICE, National Institute for Health and Care Excellence.

### Thematic review

A thematic synthesis of the 10 included studies (conducted by GL) considered implementation rates of NICE guidelines for self-harm across sectors and settings and the facilitators and barriers to adherence of each of the eight implementation priorities. 10 themes were identified: role, remit and identity; staff knowledge; resources; staff attitudes; patient characteristics; patient involvement; guideline issues; record keeping; work environment; and proforma. The most commonly identified barriers were staff knowledge (n=6), staff attitudes (n=3) and role, remit and identity (n=3). Commonly identified facilitators were use of a proforma (n=2) and role, remit and identity (n=2). More details of the barriers and facilitators identified within each priority are presented in online supplemental appendices 4 and 5.

#### Priority 1: respect, understanding and choice

##### Barrier: staff attitudes

Staff attitudes emerged as a barrier to ensuring that people who have self-harmed are treated with the same care, respect and privacy as any patient, in three studies.^35 38 40^ A self-report survey found that almost one-third of the practitioners working in a burns and plastic surgery department (n=18) were unable to agree with NICE recommendations that the treatment of self-harm injuries should be the same as for any other cause of injury.^38^ Furthermore, 10% (n=6) of staff admitted they had ‘struggled to treat patients who have self-harmed with compassion’.^38^ Negative attitudes of staff may be understated in this study, since social desirability and acquiescence bias may have influenced responses with some staff opting for amicable responses or the neutral ‘neither agree/disagree’ item, rather than indicate negativity towards this patient group.

Negative attitudes towards self-harm were echoed in a nationwide study of emergency departments, where self-harm patients were described as ‘challenging’ and labelled as ‘repeat offenders’.^40^ The inflammatory language and criminalisation of self-harm endorsed by some staff does not align with NICE guidance that self-harm patients should be viewed with compassion and afforded the same care and respect as any other patient. Attitudes were also shown to adversely affect implementation of NICE guidelines on the medical treatment of patients, with some staff (1 in 5, n=12) reporting that self-harm patients were less likely to be offered surgery.^38^

The only study that explored patients’ experiences of care^35^ reported survey results of satisfaction rates of 14 patients. Although the personal manners of staff were rated good or excellent, at least one respondent was dissatisfied with professionals’ ability to listen and understand. Together, the findings indicate that some staff hold negative attitudes towards patients who self-harm and some treatment options are denied to patients as a result. This finding is consistent with earlier research into patients’ experiences, who report discriminatory attitudes and treatment.^2 12 13 17 23^

### Priority 2: staff training

#### Barriers: staff knowledge (training accessibility); role, remit and identity (nurses and non-clinical staff); patient involvement

##### Facilitators: role, remit and identity (mental health professionals)

Seven of the 10 studies explored staff training on self-harm.^3132 34^,^38^ Accessibility of training emerged as a barrier in six of the studies.^31 32 34 35 37 38^ Two-thirds of clinicians working in a burns and plastic surgery hospital department had never undergone training on self-harm,^38^ despite most clinicians, including those who had previously had training, stating they currently wanted self-harm training. A salient finding in this study was that almost one-third of the staff admitted finding it hard to understand the reasons why patients self-harm. NICE guidelines recommend that people with lived experience are involved in the design and delivery of self-harm training;^1^ however, none of the 10 studies included in this literature review reported whether service users with a history of self-harm were involved in the training or whether the training content adhered to NICE guidelines. Also, staff training in psychosocial assessment, whereby it is recommended that mental health and emergency services jointly develop the training,^6^ was not explored. Furthermore, none of the studies explored the supervision of practitioners, despite NICE guidance advising that all staff undertaking work with individuals who self-harm have ‘regular clinical supervision in which the emotional impact upon staff members can be discussed and understood’.^6^

Low rates of staff training on self-harm were found in the three studies^35 38 39^ that measured training rates via self-report surveys of staff. Rates of training ranged from 14% to 31% (mean=26.3, SD=10.78). In a survey of an accident and emergency hospital department, just 14% (n=17) of staff indicated having received training on self-harm in the past 3 years, and less than half had ever received training that covered NICE guidelines on self-harm.^35^ This is extraordinarily low, given that self-harm is one of the most common presentations.^20^ However, many staff (57%, n=164) did not respond to a staff questionnaire about self-harm training. Although the authors^35^ report responses came from diverse staff groups and work areas, it is possible responders differed in their attitude and take-up of training compared to non-responders. In a large-scale, cross-sectoral study, spanning diverse health settings, just 31% (n=312) of the sample had received training on the assessment and management of self-harm, and for more than one-third of these respondents, the training was completed more than 5 years ago.^39^ A key finding across the studies was that nurses and non-clinical staff were less likely to have been trained in self-harm than hospital doctors and general practitioners (GPs).^36 39^ There was also a tendency for more mental health professionals to have undergone training.^39^

An emergency department reported that a proforma for self-harm consultations that was added to training sessions on self-harm, to enhance compliance with NICE guidance, was not used by locum doctors, which it was heavily reliant on^37^ indicating the addition of training sessions was a facilitator to the implementation of NICE guidelines. When whole staff training on self-harm was implemented, formal risk assessments on self-harm patients and documentation of risk factors improved, and patient self-discharge pre-assessment decreased.

### Priority 3: activated charcoal

#### Barrier: staff knowledge (lack of training)

Of the six studies involving accident and emergency departments,^33^,^3740^ only one study reported on compliance with activated charcoal provision for self-poisoning, reporting zero compliance rates for documentation.^35^

Underpinning this is a lack of staff training, that the body of literature in this review emphasises as being a key issue affecting implementation. The study reported that only six of the respondents who had received training on self-harm in the last 3 years were trained in the early management that included activated charcoal for self-poisoning.^35^

### Priority 4: triage

#### Barriers: record keeping; staff knowledge; resources (staffing, safe spaces)

Seven studies reported on the triage or initial assessment of patients who have self-harmed.^32^,^3740^

Record keeping was a barrier identified in half of the studies. Documentation of key elements of the preliminary assessment process was variable, with evidence of omissions of documentation of mental capacity,^33 36 37 40^ mental illness and level of distress,^40^ as well as willingness to remain for further assessment.^36 40^ Unclear information about ambulatory involvement in treatment recorded was also reported.^35^

Staff knowledge was identified as a barrier to triage and/or initial assessment in two studies. Nurses in general practice identified lack of knowledge of self-harm risk factors and lack of strategies to implement sensitive quality assessments as barriers.^32^ Some staff in an accident and emergency service did not assess patients’ mental capacity to consent, as they inferred that presenting to the accident and emergency service and waiting for treatment implied consent,^37^ indicative of lack of knowledge of mental capacity and consent procedures.

Resource constraints also emerged as a barrier. The only study citing evidence of safe waiting area provision for patients who had self-harmed reported this was a feature of just 2/15 accident and emergency departments across England and Scotland.^40^ Nine of the hospitals stated that mental health services were understaffed and overstretched and that one of the biggest challenges was finding one-to-one observation for high-risk patients to ensure their safety.

### Priority 5: treatment

#### Facilitators: staff knowledge and proforma

##### Barrier: staff attitudes

Staff knowledge, as a result of training, and proformas of NICE guidelines were identified facilitators to the physical treatment of self-harm in accident and emergency departments. 100% compliance rates were reported in an audit study of 834 patient notes, where all staff had received a training session, emphasising the importance of patients being offered medical treatment, regardless of whether they wished to have a psychosocial assessment.^36^ Documentation rates of the need for physical treatment rose from 38% (n=50) to 88% (n=50), following the implementation of a proforma with prompts in an audit of an accident and emergency department.^37^

Staff attitudes were a potential barrier to patients receiving appropriate physical treatment and care for their injuries. Although implementation and adherence rates were not investigated, a survey of staff attitudes in a burns and plastic surgery department revealed 10% (n=6) of staff did not agree that adequate anaesthesia and/or analgesia should be offered to patients who have self-harmed.^38^ 10% (n=6) (with 32% (n=19) neither agreeing nor disagreeing) were more likely to advocate skin grafting for accidental injuries than for similar injuries caused by self-harm. Furthermore, 14% (n=8) of staff disagreed that treatment options for self-harm injuries should be the same as any other injury cause.^38^

### Priority 6: assessment of needs

#### Barriers: record keeping; role, remit and identity; staff knowledge; staff attitudes; patient characteristics; patient involvement; guideline issues; resources

##### Facilitators: resources

Record keeping was an identified barrier to evidencing patients had undergone a comprehensive assessment of needs in five of the studies. Documentation of full mental health and social needs assessments ranged from 14% to 59% (mean=45.46, SD=16.04)^3335^,^37 40^ across studies reporting quantitative data on this priority. However, there was no evidence of patient involvement in the assessment, as recommended by NICE.

Lack of staff knowledge, wherein further assessment was dependent on scores on a risk screening tool^37^ of unreliable predictability of risk^1^ and lack of resources,^32 37^ was barriers to implementation of this priority recommendation. A qualitative study exploring general practice nurses’ use of, and adherence to national guidance for self-harm found that nurses lacked both the time and strategies needed to provide sensitive, quality consultation.^32^ Barriers involving role, remit and identity and staff attitudes also emerged. Psychosocial assessment was a lower priority compared with other targets needing to be met by nurses, and some nurses reported feeling uncertain of their remit. The complexity and generality of NICE guidelines on self-harm were also cited as a barrier (*guideline issues*) to implementation.^32^

Having access to a psychiatric liaison team (*resource*) in emergency departments was a facilitator to a full needs assessment being undertaken.^36 40^ Although patient characteristics of psychiatric history were the strongest predictor of adherence in this area,^36^ 59% (n=202) of patients whose characteristics included comorbid psychiatric illness and 44% (n=149) who had a prior history of self-harm were not assessed by the psychiatry team,^36^ even though both of these patient characteristics are associated with increased risk of suicide. The study found that 41% (n=141) of individuals who did not receive a psychosocial assessment reattended the accident and emergency department within 12 months, with a further presentation of self-harm. At this time, 48% (n=67) of the individuals received a psychosocial assessment.^36^

### Priority 7: assessment of risk

#### Facilitators: proforma; role, remit and identity; resources; work environment

##### Barriers: guideline issues; staff knowledge

Adherence rates for risk assessment ranged from 5.8% to 70% (mean=47.94, SD=36.50) in the three studies that measured this priority recommendation.^34 35 37^ With the introduction of a proforma, incorporating an adapted version of the Australian mental health triage process, as indicated in NICE (2004) guidelines,^6^ automatically printed on patient notes, as well as staff training on its usage, a follow-up audit in an emergency department showed an increase in adherence from 5.8% to 97%.^34^ Other facilitators to assessment of risk were related to role, remit and identity, including duty of care and nurses’ ability to build trust with patients. Furthermore, having access to designated safeguarding leads (*resources*) and working in a supportive environment, where advice and consultation could be readily sought, were reported as being important facilitators to assessing patient risk.^32^

Lack of staff knowledge of effective risk assessment emerged as a barrier. Two studies found that almost one in five clinicians had used self-harm risk assessment tools against NICE recommendations^32 37^ and inappropriately to determine referral for further treatment due to poor reliability.^1^ Lack of knowledge of self-harm risk factors was also a barrier to nurses being able to carry out a robust risk assessment.^32^ Guideline-related issues also emerged as a barrier with practitioners expressing concerns about missing cues of risk while referring to them.^32^

### Priority 8: psychological, psychosocial and pharmacological interventions

#### Barriers: resources; staff knowledge; role, remit and identity

Lack of resources was identified as a barrier to referral for further treatment and help for patients assessed as being at risk of further self-harm. In a study of accident and emergency departments, referral to the psychiatric liaison team was dependent on service resource within hospitals. In centres without 24-hour crisis teams, referrals to psychiatric liaison services were sometimes not made.^40^ Lack of access to resources, specifically the ability to make a referral to mental health teams, was cited as a barrier by GPs^31^ and some nurses’ lack of knowledge of self-harm risk factors, inability to refer due to their role and lack of knowledge of referral pathways also emerged as barriers to implementation in general practice.^32^

Staff knowledge of appropriate risk assessment created a further barrier to patients accessing psychological, psychosocial and pharmacological interventions. In an accident and emergency department, in England, the widespread use of the SAD PERSONS (sex, age, depression, previous attempt, ethanol abuse, rational thinking loss, social supports lacking, organised plan, no spouse, sickness) self-harm screening tool being implemented against NICE guidelines, and its score used to determine patient referral to a mental health team^37^ meant that some patients requiring referral for further treatment may not have met the threshold, due to poor predictability of screening tools on self-harm, which is the reason they are not endorsed by NICE.

## Discussion

A core theme emerging from the thematic synthesis is that frontline professionals do not receive routine training on self-harm^31 32 35 38^ and even when they do, NICE recommendations on self-harm are often not included in that training.^35^ A key recommendation is that people with lived experience of self-harm should be involved in the commissioning, planning and evaluation of services: ‘People who self-harm should be involved in the planning and delivery of training for staff’.^6^ None of the included studies explicitly explored patient and carer involvement in the development and delivery of training or input into the psychosocial assessment. In the reviewed studies, supervision and training specifically in psychosocial assessment, whereby it is recommended that mental health and emergency services jointly develop the training,^6^ was not explored. The papers reviewed indicate several impediments to NICE guideline implementation for self-harm, including lack of staff knowledge, negative staff attitudes towards patients who self-harm, lack of patient and carer involvement, lack of resources and poor record keeping. Guideline issues were also barriers, wherein the complexity and generality of the guidance created difficulties for memory retrieval and application.^31 32^ Accident and emergency departments are often the first point of contact with healthcare services for people who self-harm and are a critical gateway to mental health assessment and intervention. Research has indicated high levels of A&E admission for self-harm from people who die from suicide in the 12 months preceding death,^42^ highlighting the significance of A&E contact in suicide prevention. The findings from this rapid review showed that for many patients who had self-harmed, contact with A&E resulted in missed opportunities for robust assessment of need and risk, psychiatric liaison, care co-ordination and follow-up by GPs. Implementation of a psychosocial assessment following self-harm reduces the risk of self-harm recurrence^43^ and thus it is concerning that our review implicated all eight identified barriers to implementation in this priority area.

### Strengths and limitations of this review

By combining qualitative and quantitative studies, this review provides a comprehensive exploration of the barriers and facilitators to NICE guideline on self-harm implementation among healthcare professionals. Conducting a rapid review accelerated the process of literature synthesis with resource efficiency. While core principles of the traditional systematic review were adhered to, involving search strategy, quality assessment, transparency, data extraction and synthesis of studies, a major limitation of this rapid review is that a single reviewer searched, screened, selected, quality assessed and analysed the studies. Although several measures were taken to minimise the impact of bias (using predefined inclusion and exclusion criteria, peer and non-peer reviewed literature), the omission of non-English language articles and grey literature searches means relevant articles may have been missed. Furthermore, the search strategy used may have limited the number of articles generated. A search strategy addressing priority areas, individually, may yield a greater number of relevant articles. Thus, salient facilitators and barriers to NICE guideline implementation may not have been captured in this review. It is also recognised that the exclusion of research on clinical guidelines on self-harm other than NICE guidelines limits the generalisability of the findings. Country-specific cultural and contextual influences need to be acknowledged when interpreting the findings. The cumulative number of participants included in this review is small, affected by small sample sizes across the studies, with 8/10 studies ranging from 12 to 150.^31^,^3335 37^ The definition of self-harm varied across the included studies. One of the patient case-note audit studies did not describe how self-harm had been defined for inclusion.^33^ When inclusion criteria were documented, there were notable differences. For example, one study reviewed case notes marked ‘psychiatric self-harm’ or ‘psychiatric overdose’ ^37^ while others excluded case notes marked ‘accidental overdose’.^34 35^ Another included case notes where ‘presentation is indicative of self-harm’.^36^ The lack of specification means that it is possible that self-harm cases were not considered and conversely cases such as ‘accidental overdose’, which were explicitly screened out of other studies could have been included. Some studies excluded unconscious patients (0) and patients under the age of 16.^33 37^ While these inconsistencies introduce potential bias limiting the interpretability and generalisability of findings, it is notable that all of the barriers (negative staff attitudes towards patients who self-harm, non-specialist staff role, remit and identification, staff lacking knowledge of self-harm, lack of patient involvement, lack of resources, poor record keeping, guideline issues and complex patient characteristics) to NICE guideline implementation identified in this review is underpinned by the inclusion of high-quality studies, strengthening the credibility of these findings. Future research would benefit from standardised definitions of self-harm for inclusion and methodological consistency to enhance the reliability and applicability of the findings. Finally, the structured approach to data synthesis limited its depth and richness and subsequently may have excluded more nuanced barriers and facilitators (eg, socio-political, organisational and cultural factors) to guideline implementation outside of the priority implementation areas.

### Recommendations

The rapid review synthesis provides valuable insights into the multisystemic barriers to guideline implementation, not least lack of knowledge of the guidelines^35 37^ and understanding of role and remit in relation to implementation^32 37^ of the guidelines by frontline staff. We recommend integrating guideline prompts and proformas into electronic health records based on our findings that proformas were facilitators to implementation.^34 37^ However, innovative strategies for providing real-time guidance could also be explored. Supporting the body of evidence in the literature that patients who have self-harmed experience prejudicial attitudes from healthcare staff,^2 10 12 16 17^ this review found that stigma and discrimination adversely influence healthcare provision. Negative experiences could prevent people in life-endangering mental health crisis from seeking treatment. Coupled with the finding that frontline staff lack training on self-harm^31^,^3537^ and its positive impact on staff attitudes towards people who self-harm and NICE guideline implementation,^34 36 37^ there is a clear need for comprehensive training development and dissemination. Given the diverse settings that practitioners assessing and managing self-harm work in, with many doing shift work, we propose that e-learning should be used, that can be accessed at any time, to increase uptake. In accordance with NICE recommendations, people with lived experience of self-harm should be involved in the design and delivery (1) with content developed according to the NICE recommendations. Training should include case note documentation, given our finding that poor record keeping was a barrier to NICE guideline implementation.^3335^,^37 40^

### Recommendations for further research

Six of the 10 studies focused on accident and emergency departments.^33^,^3740^ However, only a minority of people who have self-harmed seek emergency treatment at hospital.^20^ Accordingly, research into other professionals’ practice in other areas of healthcare and across social care, education and criminal justice sectors, where people who self-harm are likely to present, is needed. All the reviewed papers examined health professionals, with hospital doctors, GPs and nurses forming most practitioners. The absence of research comprising social workers is conspicuous, given that 94% of approved mental health practitioners in England are social workers,^44^ who are routinely involved in the potential use of the Mental Health Act in the urgent assessment of people who have self-harmed. There is a need to research social workers’ implementation of NICE guidelines. Future research should also focus on children, given the high rates of self-harm in this group^45^ and lack of representation in research into NICE guidelines implementation, thus far. Also, the finding in this literature review that people using services for self-harm, their families and carers were not considered in the scope of research indicates that participatory research is needed, which considers service user and carer experiences in the development, delivery and evaluation of self-harm services, including training and experience of the psychosocial assessment.

## Conclusion

Two decades since the inception of NICE guidelines on self-harm, the paucity of research into professional awareness and implementation is remarkable. Within the dearth of available literature, this review provides important insights into the barriers and facilitators to the implementation of NICE guidelines on self-harm in the health sector. Across services, levels of awareness and implementation of NICE guidance are low. The findings of this review support previous research indicating low professional awareness levels of clinical guidelines.^21^,^23^ The aggregate findings challenge the anecdotal perception that knowledge of NICE guidelines on self-harm among staff working in emergency medicine is good.^46^ The findings have implications for staff training and supervision, in the health service and across education and social care sectors, too. In the context of rising suicide rates, with prior self-harm being its strongest predictor,^1^ it is imperative that professionals who are routinely dealing with self-harm presentations are equipped with the necessary knowledge and skills to provide safe, quality care.

## Supplementary material

10.1136/bmjopen-2024-093883 online supplemental file 1

## Data Availability

All data relevant to the study are included in the article or uploaded as supplementary information.
