# Supplementary material for: Health and social care professionals’ awareness and implementation of NICE guidelines on self-harm: a rapid review of the literature
Source: BMJ Open. 2025 Aug 19;15(8):e093883. doi: 10.1136/bmjopen-2024-093883 (PMC12366585; doi:10.1136/bmjopen-2024-093883)
Supplement: online supplemental file 1 [file bmjopen-15-8-s001.docx]

**Supplemental Material**

**Appendix 1**

**Search Strategy Terms**

| **Database** | **Search Strategy** | **Results** |
| --- | --- | --- |
| **ASSIA** | **Limiters** – date 2004-2024, English language  Summary (National Institute for Health and Care Excellence OR NICE guidelines OR NICE guid*)AND summary(self-harm* OR DSH OR self-injur* OR non-suicidal-self-injur* OR NSSI OR self-mutilat*OR self-poison* OR overdos* OR parasuicid* OR suicid*)AND summary (implementation OR implement* OR compl* OR adher* OR knowledge OR barrier* OR facilitat* OR enabl* OR challeng*) | **11** |
| **CINAHL Plus** | **Expanders**   - [**X**](https://web.p.ebscohost.com/ehost/breadbox/remove?item=expander_thesaurus&sid=a85ea65d-ca2d-4b35-84ea-c9e56a3890ab%40redis&vid=83)Apply related words - [**X**](https://web.p.ebscohost.com/ehost/breadbox/remove?item=expander_fulltext&sid=a85ea65d-ca2d-4b35-84ea-c9e56a3890ab%40redis&vid=83)Also search within the full text of the articles - [**X**](https://web.p.ebscohost.com/ehost/breadbox/remove?item=expander_enhancedsubjectprecision&sid=a85ea65d-ca2d-4b35-84ea-c9e56a3890ab%40redis&vid=83)Apply equivalent subjects   **Limiters**   - [**X**](https://web.p.ebscohost.com/ehost/breadbox/remove?item=limiter_PY&sid=a85ea65d-ca2d-4b35-84ea-c9e56a3890ab%40redis&vid=83)Publication Year: 2004-2024 - [**X**](https://web.p.ebscohost.com/ehost/breadbox/remove?item=limiter_LA1&sid=a85ea65d-ca2d-4b35-84ea-c9e56a3890ab%40redis&vid=83)English Language   TX ( National Institute for Health and Care Excellence OR NICE guidelines OR NICE guid* ) AND TX ( self-harm* OR DSH OR self-injur* OR non-suicidal-self-injur* OR NSSI OR self-mutilat*OR self-poison* OR overdos* OR parasuicid* OR suicid* ) AND XB ( implementation OR implement* OR compl* OR adher* OR knowledge OR barrier* OR facilitat* OR enabl* OR challeng* ) | **55** |
| **EMBASE via OVID** | ((National Institute for Health and Care Excellence) or NICE guidelines or NICE guid*).mp. [mp=title, abstract, heading word, drug trade name, original title, device manufacturer, drug manufacturer, device trade name, keyword heading word, floating subheading word, candidate term word] AND (self-harm* or DSH or self-injur* or non-suicidal-self-injur* or NSSI or self-mutilat*OR self-poison* or overdos* or parasuicid* or suicid*).mp. [mp=title, abstract, heading word, drug trade name, original title, device manufacturer, drug manufacturer, device trade name, keyword heading word, floating subheading word, candidate term word] AND (implementation or implement* or compl* or adher* or knowledge or barrier* or facilitat* or enabl* or challeng*).mp. [mp=title, abstract, heading word, drug trade name, original title, device manufacturer, drug manufacturer, device trade name, keyword heading word, floating subheading word, candidate term word] AND (english language and yr="2004 -Current") | **75** |
| **Medline** | ((National Institute for Health and Care Excellence) or NICE guidelines or NICE guid*).mp. [mp=title, book title, abstract, original title, name of substance word, subject heading word, floating sub-heading word, keyword heading word, organism supplementary concept word, protocol supplementary concept word, rare disease supplementary concept word, unique identifier, synonyms, population supplementary concept word, anatomy supplementary concept word] AND (self-harm* or DSH or self-injur* or non-suicidal-self-injur* or NSSI or self-mutilat*OR self-poison* or overdos* or parasuicid* or suicid*).mp. [mp=title, book title, abstract, original title, name of substance word, subject heading word, floating sub-heading word, keyword heading word, organism supplementary concept word, protocol supplementary concept word, rare disease supplementary concept word, unique identifier, synonyms, population supplementary concept word, anatomy supplementary concept word] AND (implementation or implement* or compl* or adher* or knowledge or barrier* or facilitat* or enabl* or challeng*).mp. [mp=title, book title, abstract, original title, name of substance word, subject heading word, floating sub-heading word, keyword heading word, organism supplementary concept word, protocol supplementary concept word, rare disease supplementary concept word, unique identifier, synonyms, population supplementary concept word, anatomy supplementary concept word] AND (english language and yr="2004 -Current") | **22** |
| **PsychInfo** | **Limiters** – date 2004-current, English language  ((National Institute for Health and Care Excellence) or NICE guidelines or NICE guid*).mp. [mp=title, abstract, heading word, table of contents, key concepts, original title, tests & measures, mesh word] AND (self-harm* or DSH or self-injur* or non-suicidal-self-injur* or NSSI or self-mutilat*OR self-poison* or overdos* or parasuicid* or suicid*).mp. [mp=title, abstract, heading word, table of contents, key concepts, original title, tests & measures, mesh word] AND (implementation or implement* or compl* or adher* or knowledge or barrier* or facilitat* or enabl* or challeng*).mp. [mp=title, abstract, heading word, table of contents, key concepts, original title, tests & measures, mesh word | **32** |
| **Web of Science**  - Science Citation Index Expanded (SCIE)  - Social Sciences Citation Index (SSCI)  - Arts and Humanities Citation Index (AHCI)  - Emerging Sciences Citation (ESCI)  - Conference Proceedings Citation Index (CPCI-S) | **ALL=((National Institute for Health and Care Excellence OR NICE guidelines OR NICE guid*)) AND TS=(self-harm* OR DSH OR self-injur* OR non-suicidal-self-injur* OR NSSI OR self-mutilat*OR self-poison* OR overdos* OR parasuicid* OR suicid*) AND TS=(implementation OR implement* OR compl* OR adher* OR knowledge OR barrier* OR facilitat* OR enabl* OR challeng*)** AND**(LA=(English)) AND DT=(Article) AND ((((WC=(nursing)) OR WC=(Psychiatry OR Social Work OR Medicine, General & Internal OR Education & Educational Research))) OR WC=(Health Care Sciences & Services)) OR WC=(Primary Health Care) AND DOP=(2004-01-01/2024-07-17)** | **93** |
| **Google Scholar** | NICE guideline AND self-harm AND implementation  *Search of first 10 pages of results | **10** |

**Appendix 2**

**Citations Excluded at the Stage of Full Article Review, Based on Ineligibility.**

Burbeck R, Kendall T, Lelliott P, Volans G, Baston S. Gut contamination of acutely poisoned patients: why is no one using the NICE guideline? *Emerg Med J.* 2008; 9, 619-20. doi: 10.1136/emj.2008.058693. PMID: 18723732

Courtney DB, Duda S, Szatmari P, Henderson J, Bennett K. Systematic Review and Quality Appraisal of Practice Guidelines for Self-Harm in Children and Adolescents. *Suicide Life Threat Behav.* 2019; *3*, 707-723. doi: 10.1111/sltb.12466. Epub 2018 May 2. PMID: 29722056

Lobban, F, Jones, S. Implementing clinical guidelines (or not?). *Psychology and Psychotherapy: Theory, Research and Practice. 2008;* 81, 329–330. <https://doi.org/10.1348/147608308x371778>

Mahase, E. Trusts are using unvalidated suicide risk tools against NICE guidance, researchers warn

*BMJ* 2023; 383 doi: <https://doi-org.manchester.idm.oclc.org/10.1136/bmj.p2492>

Mughal F, Burton, FM, Fletcher H. New guidance for self-harm: An opportunity not to be missed. *The British Journal of Psychiatry* 2023*; 223,* 5, 501-503. <https://dx.doi.org/10.1192/bjp.2023.113>

Pereira Carvalho N, Pierson K, Shaker-Naeeni H, Sabel E. Managing self-harm in young people presenting to the emergency department and challenges in navigating the national guidelines. *BJPsych Bull.* 2023; *47*, 4, 191-194. doi: 10.1192/bjb.2023.24. PMID: 37272611; PMCID: PMC10387412.

Pitman A, Tyrer P. Implementing clinical guidelines for self harm - highlighting key issues arising from the NICE guideline for self-harm. *Psychol Psychother.* 2008; *81*, 4, 377-97. doi: 10.1348/147608308X306897. Epub 2008 May 8. PMID: 18471348.

**Appendix 3**

**MMAT Table of Results**

| Reference | Qualitative | | | | | Quantitative  non-randomised | | | | | Quantitative  descriptive | | | | |
| --- | --- | --- | --- | --- | --- | --- | --- | --- | --- | --- | --- | --- | --- | --- | --- |
|  | 1.1 | 1.2 | 1.3 | 1.4 | 1.5 | 3.1 | 3.2 | 3.3 | 3.4 | 3.5 | 4.1 | 4.2 | 4.3 | 4.4 | 4.5 |
| **Cracknell (2015)** |  |  |  |  |  | Y | N | Y | N | Y |  |  |  |  |  |
| **Cooper et al (2008)** |  |  |  |  |  |  |  |  |  |  | Y | Y | Y | Y | Y |
| **Heyward-Chaplin et al (2018)** |  |  |  |  |  |  |  |  |  |  | Y | Y | U | U | Y |
| **Hughes et al (2007)** |  |  |  |  |  | Y | Y | Y | N | Y |  |  |  |  |  |
| **Jones et al (2007)** |  |  |  |  |  |  |  |  |  |  | Y | Y | Y | N | Y |
| **Leather et al (2020)** |  |  |  |  |  | Y | Y | N | N | Y |  |  |  |  |  |
| **Leather et al (2022)** | Y | Y | Y | Y | Y |  |  |  |  |  |  |  |  |  |  |
| **Leather et al (2023)** | Y | Y | Y | Y | Y |  |  |  |  |  |  |  |  |  |  |
| **Mullins et al (2010)** |  |  |  |  |  |  |  |  |  |  | Y | Y | Y | Y | Y |
| **Stallard et al (2022)** |  |  |  |  |  |  |  |  |  |  | Y | Y | U | N | Y |

*Included studies were qualitative, quantitative non-randomised or quantitative descriptive. None of the studies included mixed methods or randomized controlled trials.

Y = Yes

N = No

U = Unsure

**Key Descriptors:**

| 1. Qualitative | |
| --- | --- |
| 1.1 | Is the qualitative approach appropriate to answer the research question? |
| 1.2 | Are the qualitative data collection methods adequate to address the research question? |
| 1.3 | Are the findings adequately derived from the data? |
| 1.4 | Is the interpretation of results sufficiently substantiated by data? |
| 1.5 | Is there coherence between qualitative data sources, collection, analysis and interpretation? |
| 1. Quantitative non-randomized studies | |
| 3.1 | Are the participants representative of the target population? |
| 3.2 | Are measurements appropriate regarding both the outcome and intervention (or exposure)? |
| 3.3 | Are there complete outcome data? |
| 3.4 | Are the confounders accounted for in the design and analysis? |
| 3.5 | During the study period, is the intervention administered (or exposure occurred) as intended? |
| 1. Quantitative descriptive studies | |
| 4.1 | Is the sampling strategy relevant to address the research question? |
| 4.2 | Is the sample representative of the target population? |
| 4.3 | Are the measures appropriate? |
| 4.4 | Is the risk of nonresponse bias low? |
| 4.5 | Is the statistical analysis appropriate to answer the research question? |

**Appendix 4**

Themes of Barriers and Facilitators to Implementation of eight priority areas of NICE Guidelines on Self-Harm

Staff training

Activated charcoal

Triage

Treatment

Assessment of need

Assessment of risk

Psychological, psychosocial and pharmacological interventions

Respect, understanding and choice

Role, remit, and identity (mental health)

Staff Knowledge

Staff attitudes

Role, remit, and identity

Role, remit, and identity

Role, remit, and identity

Staff Knowledge

Staff attitudes

Role, remit, and identity (nurses and non-clinical)

Resources

Staff Knowledge

Staff Knowledge

Staff Knowledge

Proforma

Staff Knowledge

Resources

Resources

Patient Involvement

Record Keeping

Resources

Staff attitudes

Guideline Issues

Staff Knowledge

Proforma

Resources

Patient Characteristics

Work Environment

Patient Involvement

Guideline Issues

Record Keeping

**Appendix 5**

Codes of Barriers and Facilitators to Implementation of eight priority areas of NICE Guidelines on Self-Harm

Staff training

Activated charcoal

Triage

Treatment

Assessment of need

Assessment of risk

Psychological, psychosocial and pharmacological interventions

Respect, understanding and choice

Lack of access to training on self-harm

On-site psychiatric liaison

Role: general nursing and non-clinical staff

Role: mental health speciality

Enhanced training

Inconsistent and unclear reporting and omissions

Assumptions about capacity

Lack of Knowledge of self-harm risk factors

Lack of strategies to implement sensitive assessment

Lack of resources: staff and private safe areas

Negative staff attitudes/ disagree with guidelines

Proforma

Checkbox culture

Lack of patient involvement

Lower priority

Uncertainty of remit

Patient self-harm history

Guideline complexity

Use of risk screening tool

Distraction from patient cues of risk

Role constraints: inability to make referral

Lack of knowledge of referral pathways

Duty of care

Role: ability to build trust

Access to designed safeguarding lead

Supportive work environment

Patient psychiatric illness

Referral based on risk assessment tool
