# Supplementary material for: Health and social care professionals’ awareness and implementation of NICE guidelines on self-harm: a rapid review of the literature
Source: BMJ Open. 2025 Aug 19;15(8):e093883. doi: 10.1136/bmjopen-2024-093883 (PMC12366585; doi:10.1136/bmjopen-2024-093883)
Supplement: Supplementary file 2 [file bmjopen-2024-093883.reviewer_comments.pdf]

PEER REVIEW HISTORY

BMJ Open publishes all reviews undertaken for accepted manuscripts. Reviewers are asked to complete a checklist review form and are provided with free text boxes to elaborate on their assessment. These free text comments are reproduced below.

ARTICLE DETAILS

Title (Provisional)

Health and Social Care Professionals' Awareness and Implementation of NICE Guidelines on Self-Harm: A Rapid Review of the Literature

Authors

Lever, Gill; Dowding, Dawn; Jeyasingham, Dharman; Armitage, Chris

---

VERSION 1 - REVIEW

**Reviewer**	**1**
**Name**	**Patel, Anita**
**Affiliation**	**Imperial College London, Business School**
**Date**	**12-Dec-2024**
**COI**	**None**

This paper examines literature to identify awareness and implementation of NICE guidelines on self-harm. It is an important issue given the growing prevalence in self-harm, high prevalence among adolescents, known variations in care, stigmatising attitudes among staff etc. – in combination with the fact that there are indications that previous clinical guidelines have been sub-optimally implemented.

Ten relevant studies were identified in a rapid evidence synthesis covering the 10-year period since the relevant NICE guidelines were published. The rapid approach was taken due to the time sensitivity of needing to understand awareness and implementation of the 2022 NICE guidelines which in turn were intended to address some of the suboptimal practices that were occurring following the publication of previous guidelines. The authors acknowledged that a full systematic review may have uncovered further studies and insights. Nevertheless, they adhered to PRISMA guidelines and used the MMAT to assess study quality. They also included a wide range of databases in their searches.

Overall, the paper generates useful insights regarding barriers and facilitators to guideline implementation which are helpful for direction-setting in relations to developing solutions. Key gaps in the literature are also highlighted (e.g. regarding social care professionals, other settings in which self-harm presents, and children).

I can see no major weaknesses that the authors don't already mention themselves. Therefore, the following are simply some observations on matters that it might be helpful for the authors to expand/provide more clarity on.

1. GL's key role in generating the findings needs to be emphasised in the methods section. The authors acknowledge the use of a single reviewer as a major limitation but don't make this fact clear at the outset in the methods, which is important given the risk of bias and reduced reliability is introduced through design. Currently, there are several mentions of their role, as follows:

• The article summary states that "A limitation of this rapid review is that only one author (GL) searched, screened and quality assessed the studies".

• The methods section states that "GL tabulated data on the included articles' study characteristics (author(s), year of publication, data collection period, country, research design, setting and sample size) and reported awareness and implementation rates of NICE guidelines on self-harm."

• The results section (page 9) states that "GL applied deductive analysis (41) flexibly to both qualitative and quantitative data" and (page 10) "A thematic synthesis of the ten included studies (conducted by GL)…"

• The author statement states "GL searched, screened and quality assessed articles. GL conducted the analysis. All authors contributed to the interpretation of the results."

So it is only from the results section onwards that it becomes clear that GL generated the main results/ interpretation. Some of the methodology descriptions in the results section could move to the methods section.

2. Results, page 6, lines 50-55: The text summarising the article selection is a little unclear. What does "further processing" mean? At what stage in the selection process were 7 articles excluded? The statement "Citations are listed in appendix 2" is unclear – clarify that these were the 7 excluded ones based on eligibility. Similarly, the title for Appendix 2 needs to be expanded to state that these are citations excluded at the stage of full article review, based on ineligibility.

3. Page 9, line11: typo with reference 39 being listed twice.

4. Page 9, line 17: This sentence wasn't immediately clear; I think the comma after "alignment" could be removed to improve the clarity of this sentence.

5. In terms of methodological approach, the findings from studies are synthesised in a structured way using the priority areas identified in the 2004 NICE guidelines. This is a neat, relevant and interpretable way of capturing the findings of what I imagine were quite different studies. However, the limitations of this structured approach could also be raised e.g. some of the nuances around barriers and facilitators (e.g. cultural factors) are not really

considered, and excluding a lived experience perspective limits the extent to which patient-centred interpretations are drawn.

6. How may some of the weaknesses in the reviewed body of evidence (e.g. inconsistent inclusion criteria) have influenced the interpretation of findings from those studies for this review?

7. With regards to interpretation, systematic barriers (e.g. staff attitudes, lack of training) are drawn out clearly from the literature but can the authors suggest any specific recommendations regarding these points e.g. feasibility of training across different settings.

8. Relatively little emphasis is given to the methodological biases that the reviewed studies might present for this analysis. For example, there is a discussion about the sample size etc. but the authors could further critique if/how the moderate (versus high) quality studies impacted their interpretation of findings from those studies.

**Reviewer**	**2**
**Name**	**Griffin, Eve**
**Affiliation**	**University College Cork, National Suicide Research Foundation**
**Date**	**13-Apr-2025**
**COI**	**None**

This manuscript outlines a rapid review which examines the implementation of NICE guidelines in different healthcare settings, with a focus on identifying implementation rates, barriers and facilitators to implementation and strategies which helped implementation.

It is an interesting review which identifies important gaps and challenges in implementing components of guidelines such as NICE.

While there is a justification to taking a rapid review approach, the findings are limited to NICE guidelines and there is research in other countries of similar clinical guidelines which isn’t captured by this review, and this should be acknowledged.

Methods

The methods section needs more detail on the search process and criteria.

• Even though there is a heading with ‘inclusion and exclusion criteria’, no specific criteria are listed. It is important that all criteria for both are listed (e.g. study designs, peer-review only).

• Grey literature is mentioned via handsearching of the references, but this wouldn't constitute a grey literature search, rather an additional strategy to identify included articles. Were non-peer reviewed studies included?

• A more detailed paragraph on 'data extraction' is needed with all items mentioned. On Page 6, Line 36, there is some detail, but it doesn't specify how the data on barriers/facilitators/strategies were extracted. Perhaps the data extraction table could be included in an appendix?

• The coding framework for the barriers / facilitators used is only specified in results, this should be part of methods.

• No mention of how the data extracted were analysed? For example it's only apparent in the results section that barriers/facilitators are analysed thematically according to priority area.

• How can quantitative data be analysed deductively? Please clarify how the quantitative data were analysed.

• How were data for the review managed?

Results

It is not clear for how many studies data on barriers and facilitators were available. Only two of the studies were qualitative and most were audits. This needs to be made more explicit throughout. Is this just two studies where perhaps their results sections were analysed again?

The results section has two parts on quality assessment which could be merged.

Discussion

There were some data on ways to improve implementation / compliance (3rd aim) but these aren't really teased out? They could be a bit more.

The discussion should contain a strengths and limitations section for the review, separated out from the summary of methodological quality of the included studies.

Although there are clear recommendations in here about needing to implement guidelines such as NICE, very little of the more recent literature on this is included in the manuscript, and on recommendations for research on this.

VERSION 1 - AUTHOR RESPONSE

**Reviewer: 1**<br>**Prof. Anita Patel, Imperial College London**<br>**Comments to the Author**	
**Comments**	**Response**

1. GL's key role in generating the findings needs to be emphasised in the methods section. The authors acknowledge the use of a single reviewer as a major limitation but don't make this fact clear at the outset in the methods, which is important given the risk of bias and reduced reliability is introduced through design. Currently, there are several mentions of their role, as follows:<br>• The article summary states that "A limitation of this rapid review is that only one author (GL) searched, screened and quality assessed the studies".<br>• The methods section states that "GL tabulated data on the included articles' study characteristics (author(s), year of publication, data collection period, country, research design, setting and sample size) and reported awareness and implementation rates of NICE guidelines on self-harm."<br>• The results section (page 9) states that "GL applied deductive analysis (41) flexibly to both qualitative and quantitative data" and (page 10) "A thematic synthesis of the ten included studies (conducted by GL)…"<br>• The author statement states "GL searched, screened and quality assessed articles. GL conducted the analysis. All authors contributed to the interpretation of the results."<br>So it is only from the results section onwards that it becomes clear that GL generated the main results/ interpretation. Some of the methodology descriptions in the results section could move to the methods section.	We appreciate your careful review and feedback. We have amended the Methods section to emphasise that a single reviewer searched, screened, selected, quality assessed and analysed the studies. We have added a section on Data Extraction, Transformation and Analysis to the Methods (kindly refer to page 6) and highlighted the role of the single reviewer in this section and the 'Search Strategy' and 'Quality Assessment' sections.
2. Results, page 6, lines 50-55: The text summarising the article selection is a little unclear. What does "further processing" mean? At what stage in the selection process were 7 articles excluded? The	Thank you for your careful review and feedback. We have clarified what further processing of the articles entailed, "224 articles and abstracts were screened by a single reviewer (GL), based on the

statement “Citations are listed in appendix 2” is unclear – clarify that these were the 7 excluded ones based on eligibility. Similarly, the title for Appendix 2 needs to be expanded to state that these are citations excluded at the stage of full article review, based on ineligibility.	inclusion criteria and seventeen articles were retrieved for full text review” and the stage in the selection process that 7 articles were excluded from the review. We have also clarified that the citations listed in appendix 2 are the seven excluded articles and expanded the title for appendix 2.
3. Page 9, line11: typo with reference 39 being listed twice.	Thank you for bringing this issue to our attention. We have amended this.
4. Page 9, line 17: This sentence wasn’t immediately clear; I think the comma after “alignment” could be removed to improve the clarity of this sentence.	We appreciate your kind advice and have removed the comma.
5. In terms of methodological approach, the findings from studies are synthesised in a structured way using the priority areas identified in the 2004 NICE guidelines. This is a neat, relevant and interpretable way of capturing the findings of what I imagine were quite different studies. However, the limitations of this structured approach could also be raised e.g. some of the nuances around barriers and facilitators (e.g. cultural factors) are not really considered, and excluding a lived experience perspective limits the extent to which patient-centred interpretations are drawn.	Thank you for your helpful suggestion. We have added a section to the Discussion on ‘Strengths and Limitations of this Review” and addressed the limitations of the structured synthesis, “the structured approach to data synthesis limited its depth and richness and subsequently may have excluded more nuanced barriers and facilitators (e.g. socio-political, organisational and cultural factors) to guideline implementation outside of the priority implementation areas.” (p.18)
6. How may some of the weaknesses in the reviewed body of evidence (e.g. inconsistent inclusion criteria) have influenced the interpretation of findings from those studies for this review?	Thank you for highlighting this. We have addressed this by adding to the section of the Discussion on Strengths and Limitations of this Review:<br>“Whilst these inconsistencies introduce potential bias limiting the interpretability and generalisability of findings it is notable that all of the barriers (negative staff attitudes towards patients who self-harm, non-specialist staff role, remit and identification, staff lacking specialist knowledge of self-harm, lack of patient involvement, lack of resources, poor record keeping, guideline issues and complex patient characteristics) to NICE

	guideline implementation identified in this review are underpinned by the inclusion of high-quality studies, strengthening the credibility of these findings. Future research would benefit from standardised definitions of self-harm for inclusion and methodological consistency to enhance the reliability and applicability of the findings." (p17 – 18)
7. With regards to interpretation, systematic barriers (e.g. staff attitudes, lack of training) are drawn out clearly from the literature but can the authors suggest any specific recommendations regarding these points e.g. feasibility of training across different settings.	Thank you for your recommendation to strengthen this section. We have added a Recommendations section to the Discussion, suggesting "integrating guideline prompts and proformas into electronic health records (EHRs) based on our findings that proformas were facilitators to implementation" and "…Given the diverse settings that practitioners assessing and managing self-harm work in, with many doing shift work, we propose that e-learning should be utilised, that can be accessed at any time, to increase uptake. In accordance with NICE recommendations, people with lived experience of self-harm should be involved in the design and delivery with content developed according to the NICE recommendations. Training should also include case note documentation, given our finding that poor record keeping was a barrier to NICE guideline implementation." (p.18)
8. Relatively little emphasis is given to the methodological biases that the reviewed studies might present for this analysis. For example, there is a discussion about the sample size etc. but the authors could further critique if/how the moderate (versus high) quality studies impacted their interpretation of findings from those studies.	Thank you for bringing this issue to our attention. We have addressed this under point 6.

<table>
<tr><td colspan="2">Reviewer: 2
Dr. Eve Griffin, University College Cork
Comments to the Author:</td></tr>
<tr><td>While there is a justification to taking a rapid review approach, the findings are limited to NICE guidelines and there is research in other countries of similar clinical guidelines which isn’t captured by this review, and this should be acknowledged.</td><td>Thank you for your comment. We have acknowledged this in the Discussion:
“It is also recognised that the exclusion of research on clinical guidelines on self-harm other than NICE guidelines limits the generalisability of the findings. Country-specific cultural and contextual influences need to be acknowledged when interpreting the findings.” (p.17)</td></tr>
<tr><td>Methods
The methods section needs more detail on the search process and criteria.
• Even though there is a heading with ‘inclusion and exclusion criteria’, no specific criteria are listed. It is important that all criteria for both are listed (e.g. study designs, peer-review only).</td><td>Thank you for your kind suggestions. We have revised the inclusion and exclusion criteria to:
“Search parameters to inclusion were any original quantitative, qualitative or mixed-methods study design. There were no restrictions on sample size or peer review status. Searches were limited to English language publication, between 2004-July 2024. The cut off at 2004 ensured that only studies conducted following publication of NICE guidelines on self-harm were incorporated into the review. Studies not providing original results: systematic reviews, meta-analysis or general literature reviews. Editorials and opinion articles and research protocols were excluded. Studies conducted before 2004, and studies of non-English language publication were also ineligible.” (p.5)</td></tr>
<tr><td>• Grey literature is mentioned via handsearching of the references, but this wouldn’t constitute a grey literature search, rather an additional strategy to identify included articles. Were non-peer reviewed studies included?</td><td>Thank you for your valuable feedback. We have amended this section to “Handsearching of references of full-text articles retrieved was also performed” (p.5) and clarified that non-peer reviewed studies were included in the search strategy.</td></tr>
<tr><td>• A more detailed paragraph on ‘data extraction’ is needed with all items mentioned. On Page 6, Line 36, there is some detail, but it doesn’t specify how the</td><td>Thank you for your kind recommendation. We have added a section to the Methods on ‘Data Extraction, Transformation and Analysis’ on page 6.</td></tr>
</table>

data on barriers/facilitators/strategies were extracted. Perhaps the data extraction table could be included in an appendix?	
• The coding framework for the barriers / facilitators used is only specified in results, this should be part of methods.	Thank you for bringing this to our attention. We have now specified this in the 'Data Extraction, Transformation and Analysis' of the Methods section (p.6)
• No mention of how the data extracted were analysed? For example it's only apparent in the results section that barriers/facilitators are analysed thematically according to priority area.	We appreciate your feedback. We have clarified that, "A framework matrix of the eight key priorities for implementation of the NICE 2004 guidelines for self-harm was constructed to categorise barriers and facilitators" (p.6) in the 'Data Extraction, Transformation and Analysis' of the Methods section.
• How can quantitative data be analysed deductively? Please clarify how the quantitative data were analysed.	We have amended this section and clarified how the quantitative data were analysed:<br>"Using the convergent integrated approach recommended by the Joanna Briggs Institute (JBI) Mixed Methods Review Methodology, a single reviewer, GL, converted the quantitative data into textual descriptions (qualitative data)" (p.6)
• How were data for the review managed?	Thank you for raising this. Kindly refer to the section, 'Data Extraction, Transformation and Analysis' of the Methods section which details data management.
Results<br>It is not clear for how many studies data on barriers and facilitators were available. Only two of the studies were qualitative and most were audits. This needs to be made more explicit throughout. Is this just two studies where perhaps their results sections were analysed again?	We appreciate your valuable feedback. We have sought to clarify that data on barriers and facilitators were available for the two qualitative studies:<br>"The two qualitative studies included in this review provided data on barriers and facilitators, derived through thematic analysis. The results were analysed again and coded and themed with the cumulative data of all 10 studies." (p.10)<br>We have endeavoured to make it more explicit that only two of the studies were qualitative, "Although most studies (8) were quantitative...." (p.6)

	"Of the ten studies included in the review, two of the studies were qualitative (31-32) and eight were quantitative" (33-40).
The results section has two parts on quality assessment which could be merged.	Many thanks for this suggestion. We have merged the two parts (see p.7)
Discussion<br>There were some data on ways to improve implementation / compliance (3rd aim) but these aren't really teased out? They could be a bit more.	Thank you for your helpful suggestion. We have revised the Discussion and included a section on recommendations to improve implementation (please refer to p.18)
The discussion should contain a strengths and limitations section for the review, separated out from the summary of methodological quality of the included studies.	Thank you for your kind recommendation. This has been actioned. Please see p.17.
Although there are clear recommendations in here about needing to implement guidelines such as NICE, very little of the more recent literature on this is included in the manuscript, and on recommendations for research on this.	Thank you for your insightful comment. We have revised the Discussion and included sections on Recommendations to improve implementation and Recommendations for further research. (kindly refer to p.18)
